# Development and Validation of a Novel Stemness-Index-Related Long Noncoding RNA Signature for Breast Cancer Based on Weighted Gene Co-Expression Network Analysis

**DOI:** 10.3389/fgene.2022.760514

**Published:** 2022-02-22

**Authors:** Da Qian, Cheng Qian, Buyun Ye, Ming Xu, Danping Wu, Jialu Li, Dong Li, Bin Yu, Yijing Tao

**Affiliations:** ^1^ Department of Burn and Plastic Surgery-Hand Surgery, Changshu Hospital Affiliated to Soochow University, Changshu, China; ^2^ School of Computer Science and Engineering, Changshu Institute of Technology, Changshu, China; ^3^ Second Clinical College, Zhejiang Chinese Medical University, Hangzhou, China; ^4^ Department of Breast Surgery, Changshu Hospital Affiliated to Soochow University, Changshu, China; ^5^ Department of Breast Surgery, Changshu Hospital Affiliated to Nanjing University of Chinese Medicine, Changshu, China; ^6^ Department of Cardiology, Changshu Hospital Affiliated to Soochow University, Changshu, China

**Keywords:** breast cancer, cancer stem cells, stemness-index-related lncRNAs, WGCNA, prognosis

## Abstract

**Background:** Breast cancer (BC) is a major leading cause of woman deaths worldwide. Increasing evidence has revealed that stemness features are related to the prognosis and progression of tumors. Nevertheless, the roles of stemness-index-related long noncoding RNAs (lncRNAs) in BC remain unclear.

**Methods:** Differentially expressed stemness-index-related lncRNAs between BC and normal samples in The Cancer Genome Atlas database were screened based on weighted gene co-expression network analysis and differential analysis. Univariate Cox and least absolute shrinkage and selection operator regression analyses were performed to identify prognostic lncRNAs and construct a stemness-index-related lncRNA signature. Time-dependent receiver operating characteristic curves were plotted to evaluate the predictive capability of the stemness-index-related lncRNA signature. Moreover, correlation analysis and functional enrichment analyses were conducted to investigate the stemness-index-related lncRNA signature-related biological function. Finally, a quantitative real-time polymerase chain reaction was used to detect the expression levels of lncRNAs.

**Results:** A total of 73 differentially expressed stemness-index-related lncRNAs were identified. Next, FAM83H-AS1, HID1-AS1, HOXB-AS1, RP11-1070N10.3, RP11-1100L3.8, and RP11-696F12.1 were used to construct a stemness-index-related lncRNA signature, and receiver operating characteristic curves indicated that stemness-index-related lncRNA signature could predict the prognosis of BC well. Moreover, functional enrichment analysis suggested that differentially expressed genes between the high-risk group and low-risk group were mainly involved in immune-related biological processes and pathways. Furthermore, functional enrichment analysis of lncRNA-related protein-coding genes revealed that FAM83H-AS1, HID1-AS1, HOXB-AS1, RP11-1070N10.3, RP11-1100L3.8, and RP11-696F12.1 were associated with neuroactive ligand–receptor interaction, AMPK signaling pathway, PPAR signaling pathway, and cGMP-PKG signaling pathway. Finally, quantitative real-time polymerase chain reaction revealed that FAM83H-AS1, HID1-AS1, RP11-1100L3.8, and RP11-696F12.1 might be used as the potential diagnostic biomarkers of BC.

**Conclusion:** The stemness-index-related lncRNA signature based on FAM83H-AS1, HID1-AS1, HOXB-AS1, RP11-1070N10.3, RP11-1100L3.8, and RP11-696F12.1 could be used as an independent predictor for the survival of BC, and FAM83H-AS1, HID1-AS1, RP11-1100L3.8, and RP11-696F12.1 might be used as the diagnostic markers of BC.

## Introduction

Breast cancer (BC) is the most common malignancy among women, accounting for one-fourth of female cancer cases (Siegel et al., 2020). In 2018, there were 2.08 million new cases and 630,000 deaths worldwide (Bray et al., 2018). It is a heterogeneous disease, which can be divided into group A (luminal A type), B (luminal B type), C (HER2+ type), D (basal-like type), and E (normal-like) (Perou et al., 2000). Patients with similar clinicopathological features may have different clinical prognoses due to different gene expression patterns (Walker et al., 1997). Although the prognosis of BC has improved significantly in the past few decades due to the progress of early diagnosis and treatment, the high incidence and high mortality rate of BC still pose a major threat to human health (Early Breast Cancer Trialists' Collaborative Group et al., 2011). Therefore, accurate prediction for the prognosis of BC is very important to improve the prognosis and provide appropriate treatment for patients.

Cancer stem cells (CSCs), which have the ability of long-term self-renewal and abnormal differentiation, have been assumed to be responsible for tumorigenesis (Wang et al., 2021). It has been reported that all solid tumors contain CSCs, including BC (Al-Hajj et al., 2003), pancreatic cancer (Li et al., 2007), colorectal cancer (Dalerba et al., 2007), and ovarian cancer (Zhang et al., 2008). In addition, CSCs play an important role in tumor survival, proliferation, metastasis, and recurrence. For example, the “Driver Network” regulatory molecules FoxM1 and mybl2, which are involved in cell proliferation, can be used as potential biomarkers and therapeutic targets for non-small cell lung cancer (Ahmed, 2019). Increasing evidence has revealed that transcriptomic and epigenomic features are related to cancer stemness (Young, 2011; Bradner et al., 2017). Malta et al. developed two independent stemness indices, including DNA methylation-based stemness index and messenger RNA expression-based stemness index (mRNAsi), based on molecular profiles from The Cancer Genome Atlas (TCGA) database (https://portal.gdc.cancer.gov/) using an innovative one-class logistic regression machine-learning algorithm (Sokolov et al., 2016). DNA methylation-based stemness index and mRNAsi were derived using a one-class logistic regression machine-learning algorithm trained on stem cell, embryonic stem cell, induced pluripotent stem cell classes, and their differentiated ecto-, meso-, and endoderm progenitors and ranges from low (zero) to high (one) stemness. (Sokolov et al., 2016). Moreover, epigenetic regulation-based stemness index (EREG-mRNAsi) is a stemness index generated using a set of stemness-related genes that were regulated by DNA methylation (Malta et al., 2018). An increasing number of studies have suggested that mRNAsi and EREG-mRNAsi are important indexes to evaluate the overall stemness of CSCs (Wang et al., 2021). Moreover, recent studies have suggested a correlation between stemness-index-related genes and the survival and prognosis of cancer patients in all TCGA tumors (Malta et al., 2018). For instance, Zhang et al. (2020) found that the stemness-index-associated signature, including seven stemness-index-related genes, can predict the prognosis of primary lower-grade glioma. In addition, Chen X. et al. (2020) identified 17 key stemness-index-related genes and constructed a nine-gene risk mode to predict the disease outcome of gastric cancer patients.

Long noncoding RNA (lncRNA) is a kind of noncoding RNA with a length of 200-bp–100 KB. It is mainly produced by destroying the structure of protein-coding genes (Fedor et al., 2013). It exists in the cytoplasm and interacts with other molecules in the cell to regulate the physiological and biochemical processes in organisms (Fan and Hu, 2016). The role of lncRNA in BC has been gradually revealed. LncRNA-encoded polypeptide ASRPS inhibits triple-negative BC angiogenesis (Wang et al., 2020). LncRNA TINCR promotes chemoresistance and epithelial–mesenchymal transition in BC through targeting microRNA-125b (Dong et al., 2019). More importantly, previous studies have suggested that lncRNA has potential implications in facilitating the tumorigenesis and stemness of cancer. For example, lncRNA LOXL1-AS1 can facilitate the tumorigenesis and stemness of gastric carcinoma *via* the regulation of the miR-708-5p/USF1 pathway (Sun et al., 2019). LncRNA TUG1 facilitates proliferation, invasion, and stemness of ovarian cancer cells (Zhan et al., 2020). However, the prognostic and diagnostic value of stemness-index-related lncRNAs in BC has been few reported.

The present study aims to screen stemness-index-related lncRNAs in BC based on weighted gene co-expression network analysis (WGCNA) and construct a stemness-index-related lncRNA signature to predict the prognosis of BC patients. Moreover, we also explored the diagnostic values and biological functions of lncRNAs in stemness-index-related lncRNA signature. It is of great significance in improving not only the clinical diagnosis level of BC but also the prognosis of BC patients and providing the basis for the treatment of BC.

## Materials and Methods

### Data Sources

The expression profiles of lncRNAs and mRNAs from 1,072 BC patients and 99 adjacent normal tissues and the clinical information of BC patients were downloaded from TCGA database. The mRNAsi and EREG-mRNAsi data of each BC patient were obtained from previous literature (Sokolov et al., 2016). After eliminating BC patients missing overall survival (OS) and the stemness index information, a total of 1,050 BC patients were retained for further analyses. Moreover, the microarray expression profile of the GSE20685 (including 327 BC patients with OS) dataset was acquired from the Gene Expression Omnibus database (https://www.ncbi.nlm.nih.gov/geo/) as a validation set.

### Identification of Stemness-Index-Related Module and Long Noncoding RNAs Based on Weighted Gene Correlation Network Analysis

To identify stemness-index-related lncRNAs, WGCNA was carried out *via* the “WGCNA” package in R based on the expression profile of lncRNAs of 1,050 BC patients from TCGA database (Langfelder and Horvath, 2008). Firstly, samples' cluster analysis was conducted with the hclust function to remove the outlier samples. Next, the softPower = sft$powerEstimate command was used to select an optimal soft threshold to ensure that interactions among lncRNAs conform to the scale-free distribution to the maximum extent (Kenny, 2019). In addition, we calculated the adjacency among lncRNAs, and the adjacency matrix was used to construct a co-expression network by calculating topological overlap matrix (TO), which was then hierarchically clustered with (1-TO) as a distance measure. Furthermore, modules were identified by a dynamic shear tree algorithm, with the parameters MEDissThres set to 0.2 and minModuleSize set to 30 (Zhan et al., 2020). Finally, the key module was selected for further analyses based on the correlation coefficients between modules and the traits, and the lncRNAs in the key module were defined as stemness-index-related lncRNAs.

### Identification of Differentially Expressed Stemness-Index-Related Long Noncoding RNAs

Firstly, we extracted the expression matrix of stemness-index-related lncRNAs from the BC and normal samples in TCGA database. Next, the “limma” package in R was selected to perform the differential expression analysis (Ritchie et al., 2015), and lncRNAs with adj < 0.05 and fold change > 1.5 were selected as differentially expressed stemness-index-related lncRNAs.

### Construction and Validation of a Stemness-Index-Related Long Noncoding RNA Signature Associated With the Survival of Breast Cancer Patients

To construct a signature based on stemness-index-related lncRNAs, univariate Cox regression analysis was used to screen the prognosis-related lncRNAs from the differentially expressed stemness-index-related lncRNAs by “survival” package in R in TCGA database. The results of univariate Cox regression analysis were shown by the forestplot plotted using the “forestplot” package in R. Next, lncRNAs with *p* < .1 were used to perform the least absolute shrinkage and selection operator (LASSO) regression analysis through the “glmnet” package in R for constructing the optimal stemness-index-related lncRNA signature in TCGA database (Friedman et al., 2010). Subsequently, a stemness-index-related lncRNA signature was established based on the expression values of lncRNAs and corresponding coefficients obtained by LASSO regression analysis. Thus, the stemness-index-related lncRNA signature was established according to the expression values of these six lncRNAs and the corresponding coefficient derived from the LASSO Cox regression analysis. Namely, the risk score of each patient, which was calculated as follows: risk score = (expression of lncRNA 1 × coefficient of lncRNA 1) + (expression of lncRNA 2 × coefficient of lncRNA 2) + … + (expression of lncRNA n × coefficient of lncRNA n), was the sum of the products of the expression values of these six lncRNAs and their respective LASSO coefficients. Therefore, BC patients in TCGA database and GSE20585 dataset were, respectively, stratified into the high-risk and low-risk groups based on the median value of the risk scores in all BC patients. Finally, Kaplan–Meier (K-M) survival analyses using the “survminer” package in R and the log-rank test were performed to compare the OS of patients in the high-risk and low-risk groups. Time-dependent receiver operating characteristic (ROC) curves were plotted to investigate the prediction accuracy for prognosis prediction of the stemness-index-related lncRNA signature, and the area under the curve for 1-, 3-, and 5-year OS was calculated through the “survivalROC” package in R (Heagerty and Zheng, 2005).

### Stratified Survival Analysis

To further investigate whether the stemness-index-related lncRNA signature could apply in different clinicopathological characteristics, we also investigated the OS between the high-risk and low-risk groups based on the median value of the risk scores in different clinical features using the “survminer” package in R.

### Independent Prognostic Analysis

To investigate whether the stemness-index-related lncRNA signature could act as an independent prognostic prediction factor, the stemness-index-related lncRNA signature and other clinical features were merged to screen independently prognostic prediction factor *via* univariate and multivariate Cox regression analyses in TCGA database. Similarly, the results of univariate and multivariate Cox regression analyses were shown by the forestplots plotted using the “forestplot” package in R.

### Identification of Stemness-Index-Related Long Noncoding RNA Signature Related to Biological Function

Firstly, we identified the differentially expressed genes (DEGs) between the high-risk and low-risk groups using the “limma” package in R, with the cutoff values of adj.P.Val < 0.05 and Fold Change > 1.5 (Ritchie et al., 2015). Next, Gene Ontology (GO) annotation (including biological process, molecular function, and cellular component) and Kyoto Encyclopedia of Genes and Genomes (KEGG) pathway enrichment analysis were performed to explore the biological function of DEGs using the “clusterProfiler” package in R (Yu et al., 2012), and adj.P.Val < 0.05 was considered significant enrichment. Finally, the “ggplot2” package in R was used to plot bubble diagrams to show the results of enrichment analyses. Moreover, single-sample gene set enrichment analysis, which computed an enrichment score representing the degree to which genes in a particular gene set were coordinately up- or downregulated within a single sample, was used to investigate the immune cell infiltration between the high-risk and low-risk groups using GSVA R package-based immune cell-related gene sets (Barbie et al., 2009).

### Identification of Long Noncoding RNA-Related Protein-Coding Gene and Function Enrichment Analysis

To investigate the potential regulatory mechanisms of lncRNAs in the stemness-index-related lncRNA signature, Pearson's correlation analysis was performed to identify protein-coding genes related to lncRNAs in the stemness-index-related lncRNA signature in BC patients from TCGA database using psych v.2.0.12 package in R, with the parameters set as |R| > 0.5 and *p* < 0.01. Moreover, the “VennDiagram” package in R was used to recognize the overlapping genes among each lncRNA-related gene. Furthermore, the correlation network and mechanism were visualized using Cytoscape version 3.8.0 and “Ggalluvial” package in R. Finally, KEGG pathway analysis of protein-coding genes related to each lncRNA in the stemness-index-related lncRNA signature was conducted using “clusterProfiler package” in R, and *p* < .05 was considered significant enrichment.

### Investigation of the Diagnostic Value of Long Noncoding RNAs in the Stemness-Index-Related Long Noncoding RNA Signature

Firstly, we examined the expression levels of lncRNAs in the stemness-index-related lncRNA signature in TCGA database. Next, ROC curves were plotted to show the performance of lncRNAs for distinguishing BC and normal samples (Heagerty and Zheng, 2005).

### Validation of the Expression of Long Noncoding RNAs in the Stemness-Index-Related Long Noncoding RNA Signature by Quantitative Real-Time Polymerase Chain Reaction

The normal breast epithelial cell line MCF-10A and human BC cell lines MCF-7, T47D, ZR-75-1, MDA-MB-231, and MDA-MB-468 were purchased from the American Tissue Culture Collection (Rockville, MD, USA) and were maintained in our lab. The MCF-10A cells were cultured in MEB medium (Lonza Group Ltd., Basel, Switzerland) with 100 U/ml penicillin (Sigma, St. Louis, MO, USA), 100 U/ml streptomycin (Sigma), 20 ng/ml human epidermal growth factor (Lonza Group Ltd.), 0.5 μg/ml hydrocortisone (Lonza Group Ltd.), 100 ng/ml cholera toxin (Lonza Group Ltd.), 10 μg/ml human insulin (Lonza Group Ltd.), and 5% horse serum (Sigma). The MCF-7 cells were maintained in DME medium (Gibco, Invitrogen Corporation, NY, USA) with 100 U/ml penicillin, 100 μg/ml streptomycin, 10 μg/ml human insulin (Sigma), 10% fetal bovine serum (Hyclone, Logan, UT, USA). The T47D cells were maintained in RPMI-1640 medium (Gibco, Invitrogen Corporation) with 100 U/ml penicillin, 100 μg/ml streptomycin, 0.2 U/ml bovine insulin (Sigma), and 10% fetal bovine serum. The ZR-75-1 cells were maintained in RPMI-1640 medium with 100 U/ml penicillin, 100 μg/ml streptomycin, and 10% fetal bovine serum. The MDA-MB-231 and MDA-MB-468 cells were cultured in Leibovitz's L-15 medium (Gibco, Invitrogen Corporation) with 100 U/ml penicillin, 100 μg/ml streptomycin, and 10% fetal bovine serum.

The TRIzol reagent (Thermo, MA, USA) was used to extract the total RNA of cells according to the manufacturer's instructions. Next, total RNA was reverse transcribed into complementary DNA using the iScript cDNA Synthesis Kit (Bio-Rad, Hercules, CA, USA) based on the manufacturer's procedure. Moreover, quantitative real-time polymerase chain reaction (PCR) was performed using SYBR Green Premix Ex Taq (Takara, Japan) and the Applied Biosystems 7,500 Real-time PCR System (Applied Biosystems, Inc., Carlsbad, CA, USA). Finally, the relative expression level of each lncRNA was calculated using the 2-^ΔΔCt^ method, ΔΔCt = (CtRNA—Ctβ-actin) BC cells—(CtRNA—Ctβ-actin) normal cells, and fold change = 2^−ΔΔCt^. Primer sequences and annealing temperatures of quantitative real-time PCR could be found in Table 1

### Statistical Analysis

Statistical analyses in the present study were performed through R software. The t-test and log-rank test compared the differences between different groups. The expression levels of each lncRNA between normal cells and BC cells were compared with one-way ANOVA and Tukey's test. *p* < .05 is considered statistically significant unless otherwise noted.

## Results

### Identification of Stemness-Index-Related Module and Long Noncoding RNAs Based on Weighted Gene Co-Expression Network Analysis

**FIGURE 1:**
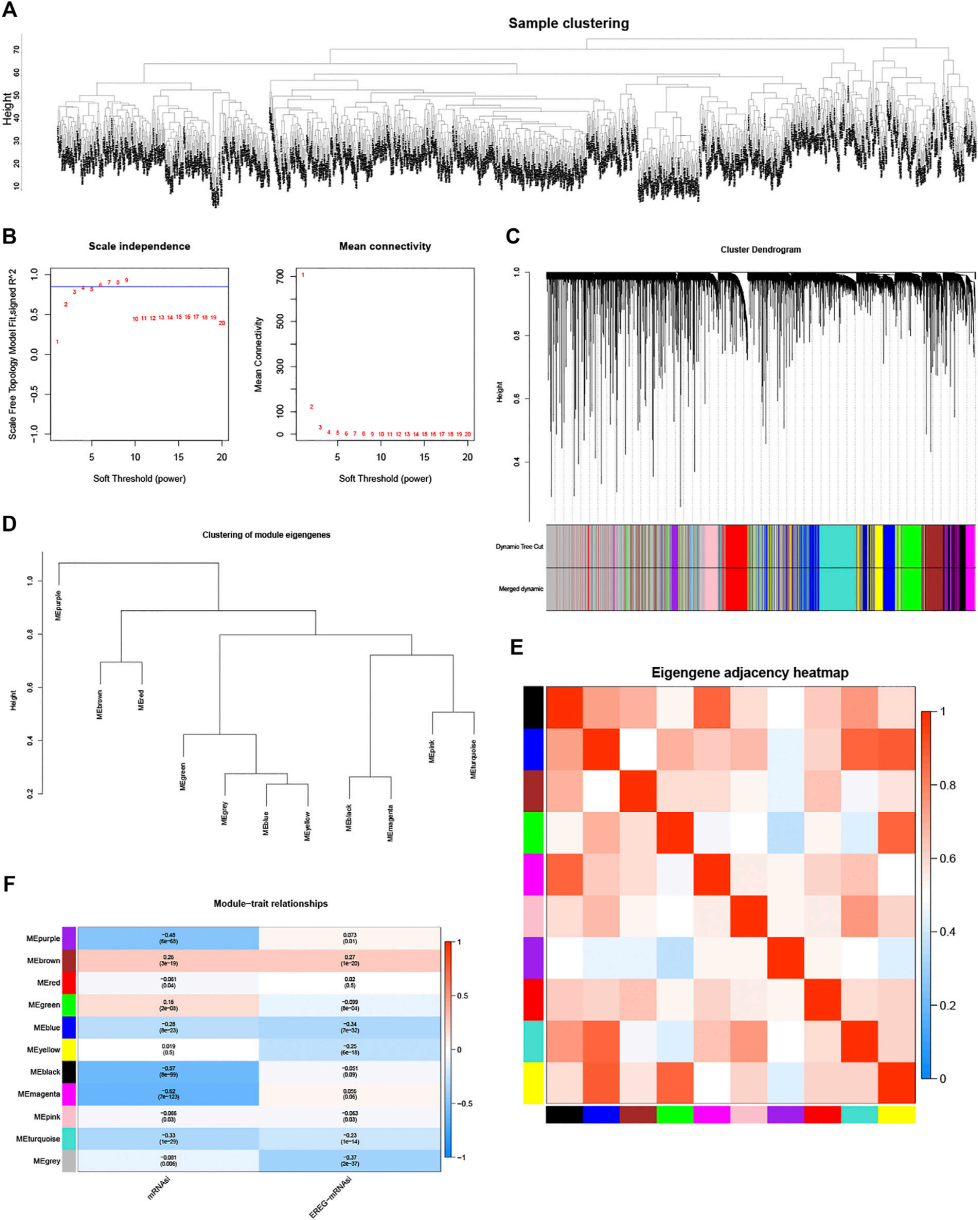
Identification of stemness-index-related module and lncRNAs based on WGCNA. Samples clustering analysis to remove outliers **(A)**, determination of soft threshold and inspection of scale-free network **(B)**, 11 modules were identified and presented in different colors by setting MEDissThres as 0.2 and minModuleSize as 30 **(C)**, 11 modules were grouped into four clusters by correlation analyses **(D)**, purple module has lowest correlation with other modules **(E)**, and magenta module was most significantly negatively correlated with mRNAsi (*p* < .05 and correlation coefficient = −0.62) **(F)**.

After sample cluster analysis, genes in 1,050 BC samples were selected to construct a weighted gene co-expression network (Figure 1A). Subsequently, soft threshold selection analysis suggested that *β* = 6 (scale-free R2 = 0.85) was optimal soft thresholds (Figure 1B). Moreover, by setting MEDissThres as 0.2 and minModuleSize as 30, a total of 11 modules were identified and presented in different colors (Figure 1C). Correlation analyses between any two-module revealed that 11 modules were grouped into four clusters (Figure 1D). Moreover, the correlation heatmap of modules also suggested that the purple module has the lowest correlation with other modules (Figure 1E). Finally, correlation analysis suggested that the magenta module was the most significantly negatively correlated with mRNAsi (Figure 1F, *p* < .05 and correlation coefficient = -0.62). Thus, the magenta module was defined as a stemness-index-related module, and 299 lncRNAs in this module were defined as stemness-index-related lncRNAs.

### Identification of Differentially Expressed Stemness-Index-Related Long Noncoding RNAs

To screen differentially expressed stemness-index-related lncRNAs, we extracted the expression matrix of 299 stemness-index-related lncRNAs from the BC and normal samples in TCGA database. Under the cutoff value of adj.P.Val < 0.05 and fold change > 1.5, a total of 73 lncRNAs, including seven upregulated lncRNAs and 66 downregulated lncRNAs in BC samples compared with normal samples, were identified as differentially expressed stemness-index-related lncRNAs (Supplementary Figure S1, Supplementary Material S1).

### Construction and Validation of a Stemness-Index-Related Long Noncoding RNA Signature Associated With the Survival of Breast Cancer Patients

**FIGURE 2:**
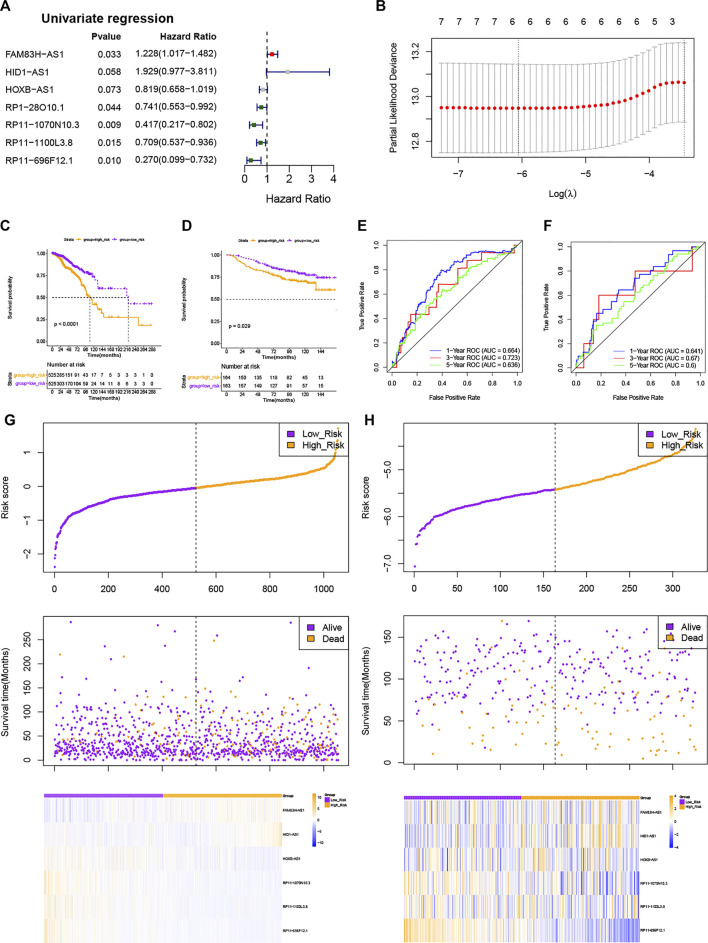
Construction and validation of a stemness-index-related lncRNA signature associated with survival of BC patients. Results of univariate Cox regression analysis **(A)** and LASSO Cox regression analysis **(B)**, Kaplan–Meier survival analysis between high-risk and low-risk groups in TCGA database **(C)** and GSE20585 dataset **(D)**, ROC curve evaluated efficiency of stemness-index-related lncRNA signature for predicting 1-, 3-, and 5-year OS in TCGA database **(E)** and GSE20585 dataset **(F)**, and lncRNAs expression profiles, risk scores distribution, and patients' survival status in TCGA database **(G)** and GSE20585 dataset **(H)**.

**FIGURE 3:**
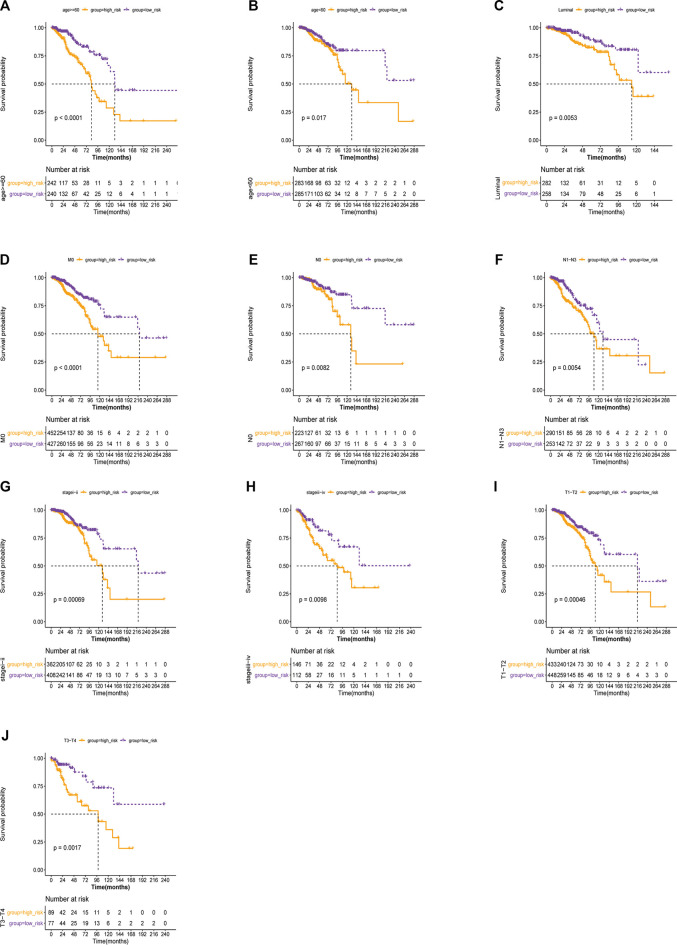
Kaplan–Meier survival stratifcation analyses in TCGA database based on stemness-index-related lncRNA signature. Age > = 60 years **(A)**, age < 60 years **(B)**, female **(C)**, M0 **(D)**, N0 **(E)**, N1–N3 **(F)**, stages i–ii **(G)**, stages iii-iv **(H)**, T1–T2 **(I)**, T3–T4 **(J)**.

To establish a stemness-index-related lncRNA signature, univariate Cox regression analysis was carried out to identify the prognostic lncRNAs in BC patients from the differentially expressed stemness-index-related lncRNAs in TCGA database. As shown in Figure 2A, univariate Cox regression analysis revealed that RP11-1070N10.3, RP11-696F12.1, RP11-1100L3.8, FAM83H-AS1, RP1-28O10.1, HID1-AS1, and HOXB-AS1 were related to the prognosis of BC patients at the cutoff value of *p* < .1. Next, LASSO Cox regression analysis suggested that FAM83H-AS1, HID1-AS1, HOXB-AS1, RP11-1070N10.3, RP11-1100L3.8, and RP11-696F12.1 were retained to construct a stemness-index-related lncRNA signature based on the optimal lambda value (Figure 2B). Thus, the risk score of each patient was calculated as follows: Risk score = expression value of FAM83H-AS1 × 0.0791 + expression value of HID1-AS1 × 1.1969 + expression value of HOXB-AS1 × (−0.1696) + expression value of RP11-1070N10.3 × (-0.7021) + expression value of RP11-1100L3.8 × (-0.2640) + expression value of RP11-696F12.1 × (−0.8372). At the same time, the coefficient also suggested that FAM83H-AS1 and HID1-AS1 were risk factors (hazard ratio > 1), but the other four lncRNAs were protective factors (hazard ratio < 1), which was consistent with the results of the K-M survival analysis (Supplementary Figure S2). Therefore, BC patients in TCGA database were divided into the high-risk and low-risk groups based on the median value of risk scores. In addition, K-M survival analysis suggested that patients in the high-risk group showed significantly lower OS than those in the low-risk group (Figure 2C). As illustrated in Figure 2E, the area under the curve values for predicting the 1-, 3-, and 5-year survival were 0.664 at 1 year, 0.723 at 3 years, and 0.636 at 5 years, suggesting that stemness-index-related lncRNA signature could predict the 1-, 3-, and 5-year survival of BC patients well. Consistently, the high-risk group included more dead samples than the low-risk group (Figure 2G). Furthermore, the analyses of the expression levels for these six lncRNAs between the high-risk and low-risk groups also suggested that FAM83H-AS1 and HID1-AS1 were risk factors, but other lncRNAs were protective factors (Figure 2G), which was consistent with the results of the LASSO Cox regression analysis. On the other hand, K-M survival, ROC analysis, these six lncRNA expression profiles, the risk scores distribution, and patients' survival status analyses in the GSE20585 dataset also showed the same results as TCGA database (Figures 2D,F,H). Furthermore, the stratified survival analysis in TCGA database also suggested that the stemness-index-related lncRNA signature also could predict the OS in different clinical features, including age, pathological stage, and luminal subtype (Figure 3). To sum up, these results indicated that the stemness-index-related lncRNA signature based on FAM83H-AS1, HID1-AS1, HOXB-AS1, RP11-1070N10.3, RP11-1100L3.8, and RP11-696F12.1 could predict the survival of BC patients well.

### Stemness-Index-Related Long Noncoding RNA Signature Was an Independently Prognostic Factor in Breast Cancer

**FIGURE 4:**
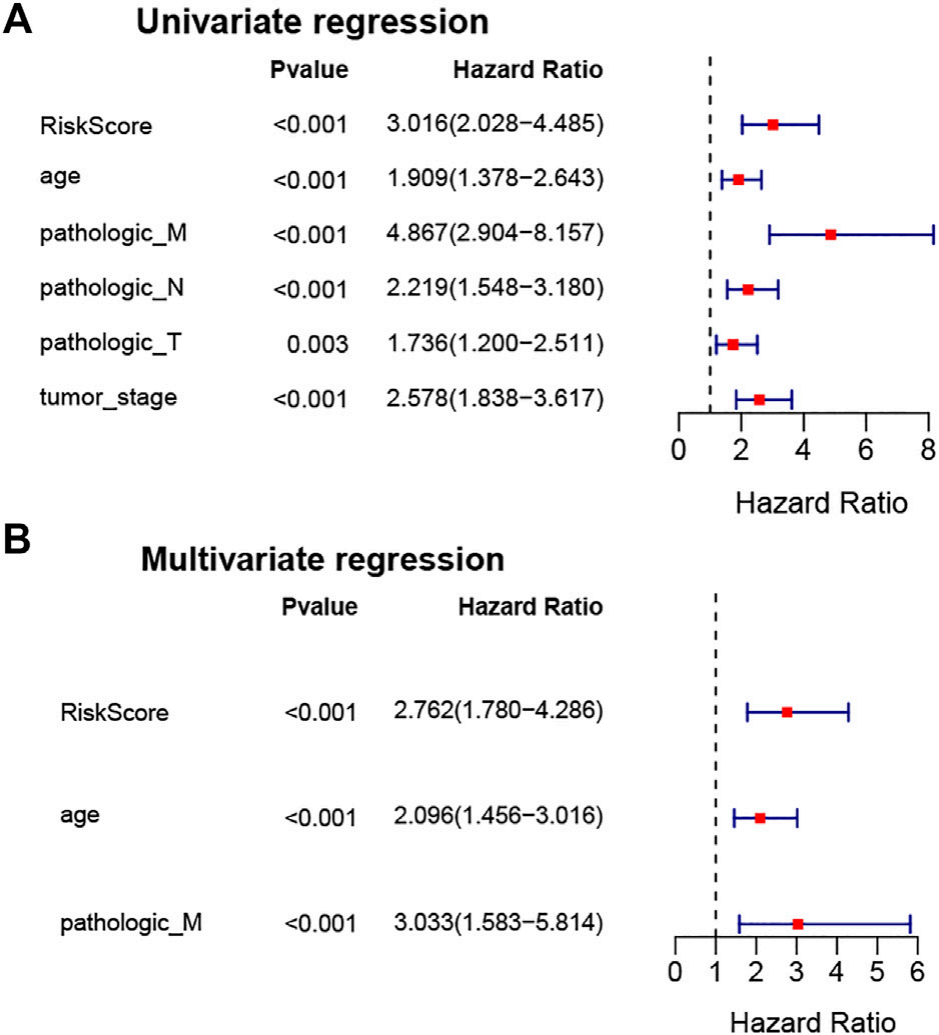
Stemness-index-related lncRNA signature was an independent prognostic factor in BC. Univariate Cox regression analysis **(A)** and multivariate Cox regression analysis **(B)** to identify independent prognostic factors from stemness-index-related lncRNAs and other clinicopathological characteristics in TCGA database.

To verify whether the stemness-index-related lncRNA signature could be used as an independent prognostic factor in BC patients, univariate and multivariate Cox regression analyses were performed to identify independent prognostic factors from the clinicopathological characteristics and the stemness-index-related lncRNA signature. Surprisingly, univariate Cox regression analysis demonstrated that the stemness-index-related lncRNA signature, age, pathological M stage, pathological N stage, pathological T stage, and pathological tumor stage were associated with the OS in BC patients (*p* < .05, Figure 4A). Moreover, multivariate Cox regression analysis confirmed that the stemness-index-related lncRNA signature, age, and pathological M stage could act as independent prognostic factors for predicting the prognosis of BC patients (*p* < .05, Figure 4B). Thus, the stemness-index-related lncRNA signature was an independent prognostic factor in BC.

### Functional Annotation of the Stemness-Index-Related Long Noncoding RNA Signature

**FIGURE 5:**
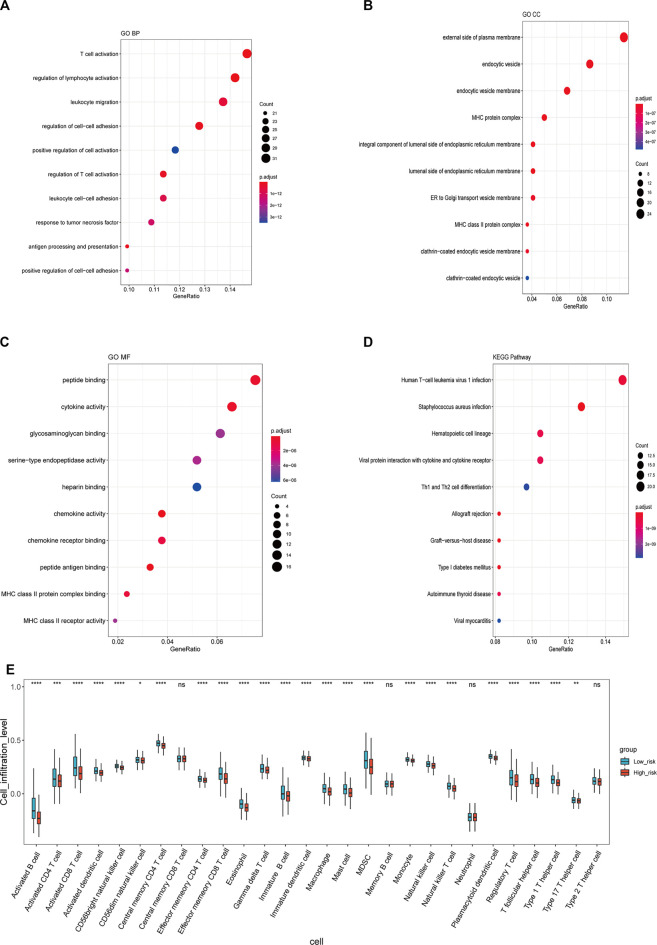
Functional annotation of stemness-index-related lncRNA signature. GO-Biological processes **(A)**, GO-Cellular component **(B)**, GO-Molecular function **(C)**, and KEGG pathway enrichment analysis **(D)**. Immune cell infiltration between high- and low-risk groups **(E)**. GO, Gene Ontology; KEGG, Kyoto Encyclopedia of Genes and Genomes.

To investigate the GO functions and KEGG pathways related to the stemness-index-related lncRNA signature, GO annotation and KEGG pathway enrichment analysis were performed to explore the biological function of DEGs between the high-risk and low-risk groups. Firstly, 236 DEGs, including 24 upregulated and 212 downregulated, were identified (Supplementary Figure S3, Supplementary Material S2). Moreover, for biological processes, DEGs were mainly involved in T-cell activation, regulation of lymphocyte activation, and leukocyte migration (Figure 5A). For cellular components, DEGs were mainly related to the external of the plasma membrane, endocytic vesicle, and endocytic vesicle membrane (Figure 5B). For molecular function, DEGs were mainly associated with peptide binding, cytokine activity, and glycosaminoglycan binding (Figure 5C). Furthermore, KEGG pathway enrichment analysis suggested that DEGs were mainly related to human T-cell leukemia virus one infection, *Staphylococcus aureus* infection, hematopoietic lineage, viral protein interaction with cytokine and cytokine receptor, and Th1 and Th2 cell differentiation (Figure 5D). Finally, we also found that the infiltrations of a majority of the immune cell were significantly different in the high- and low-risk groups (Figure 5E). Therefore, these six lncRNAs in the stemness-index-related lncRNA signature might affect the CSC by regulating the composition of immune cells in the tumor microenvironment (TME) of BC.

### Identification of Long Noncoding RNA-Related Protein-Coding Genes and Functions Enrichment

**FIGURE 6:**
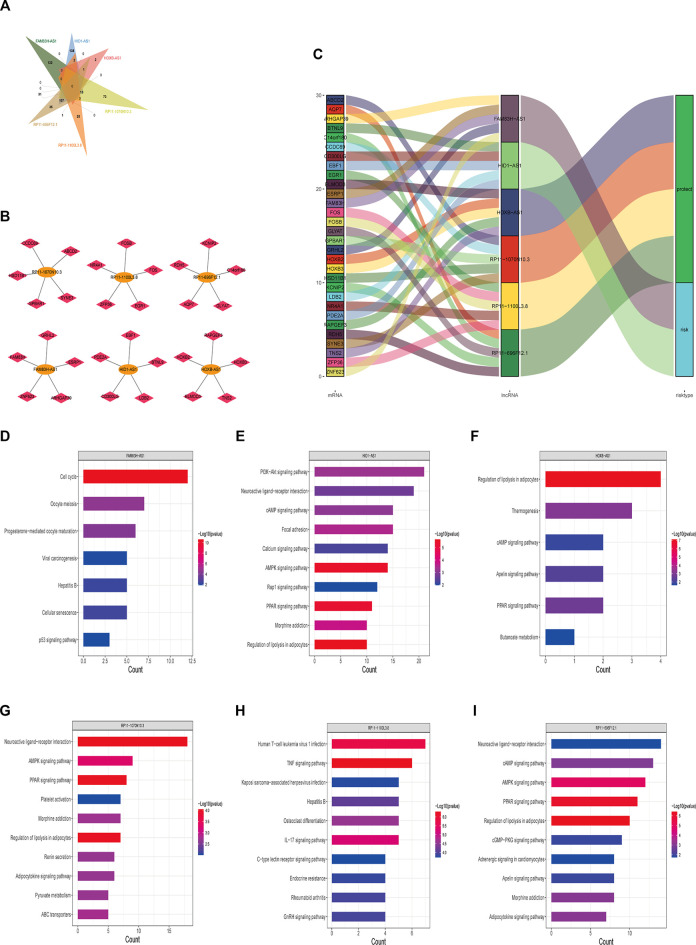
Potential regulatory mechanisms of lncRNAs in stemness-index-related lncRNA signature. Venn diagram of protein-coding gene associated with all of six lncRNAs **(A)**, interaction network of five most relevant protein-coding genes and each lncRNA **(B)**, and Sankey diagram showed five most relevant protein-coding genes of each lncRNA **(C)**. KEGG pathway enrichment analysis of each lncRNA-related protein-coding gene. FAM83H-AS1 **(D)**, HID1-AS1 **(E)**, HOXB-AS1 **(F)**, RP11-1070N10.3 **(G)**, RP11-1100L3.8 **(H)**, and RP11-696F12.1 **(I)**. KEGG, Kyoto Encyclopedia of Genes and Genomes.

To further explore the correlation between lncRNAs in the stemness-index-related lncRNA signature, we carried out the correlation analysis between these six lncRNAs and the protein-coding genes based on the expression matrix of six lncRNAs and the protein-coding genes from the BC patients in TCGA database. The results of the correlation analysis revealed that 132 protein-coding genes were related to FAM83H-AS1, 493 protein-coding genes were related to HID1-AS1, 13 protein-coding genes were related to HOXB-AS1, 340 protein-coding genes were related to RP11-1070N10.3, 37 protein-coding genes were related to RP11-1100L3.8, and 408 protein-coding genes were related to RP11-696F12.1 (Supplementary Material S3). Interestingly, none of the protein-coding genes was associated with all of these six lncRNAs (Figure 6A), indicating these six lncRNAs might independently influence the CSC of BC. Moreover, we also constructed a lncRNA protein-coding gene network based on the five most relevant protein-coding genes of each lncRNA. As shown in Figure 6B, FAM83H-AS1 was associated with FAM83H, GRHL2, ESRP1, ARHGAP39, and ZNF623, HID1-AS1 was associated with PDE2A, EBF1, BTNL9, LDB2, and CD300LG, HOXB-AS1 was associated with HOXB2, RAPGEF3, HOXB3, TNS2, and ELMOD3, RP11-1070N10.3 was associated with CCDC69, ABCD2, SYNE3, GPBAR1, and HSD11B1, RP11-1100L3.8 was associated with FOSB, FOS, EGR1, ZFP36, and NR4A1, and RP11-696F12.1 was associated with RDH5, KCNIP2, C14orf180, GLAYT, and AQP7. The Sankey diagram showed that the five most relevant protein-coding genes of each lncRNA may be related to one or more lncRNAs (Figure 6C). Finally, the KEGG pathway enrichment analysis of each lncRNA-related genes suggested that FAM83H-AS1-related genes were involved in cell cycle, oocyte meiosis, progesterone-mediated oocyte maturation, and viral carcinogenesis (Figure 6D); HID1-AS1-related genes were involved in PI3K-Akt signaling pathway neuroactive ligand–receptor interaction, cAMP signaling pathway, AMPK signaling pathway, Rap1 signaling pathway, and PPAR signaling pathway (Figure 6E); HOXB-AS1-related genes were involved in regulation of lipolysis in adipocytes, cAMP signaling pathway, apelin signaling pathway, and PPAR signaling pathway (Figure 6F); RP11-1070N10.3-related genes were involved in neuroactive ligand–receptor interaction, AMPK signaling pathway, PPAR signaling pathway, and adipocytokine signaling pathway (Figure 6G); RP11-1100L3.8-related genes were involved in human T-cell leukemia virus one infection, TNF signaling pathway, IL-17 signaling pathway, C-type lectin receptor signaling pathway, and GnRH signaling pathway (Figure 6H); and RP11-696F12.1-related genes were involved in neuroactive ligand–receptor interaction, cAMP signaling pathway, AMPK signaling pathway, PPAR signaling pathway, cGMP-PKG signaling pathway, apelin signaling pathway, and adipocytokine signaling pathway (Figure 6I). Therefore, it was speculated that FAM83H-AS1, HID1-AS1, HOXB-AS1, RP11-1070N10.3, RP11-1100L3.8, and RP11-696F12.1 might play key roles in BC by regulating the AMPK signaling pathway, PPAR signaling pathway, and cGMP-PKG signaling pathway.

### Investigation of the Diagnostic Value of Long Noncoding RNAs in the Stemness-Index-Related Long Noncoding RNA Signature

**FIGURE 7:**
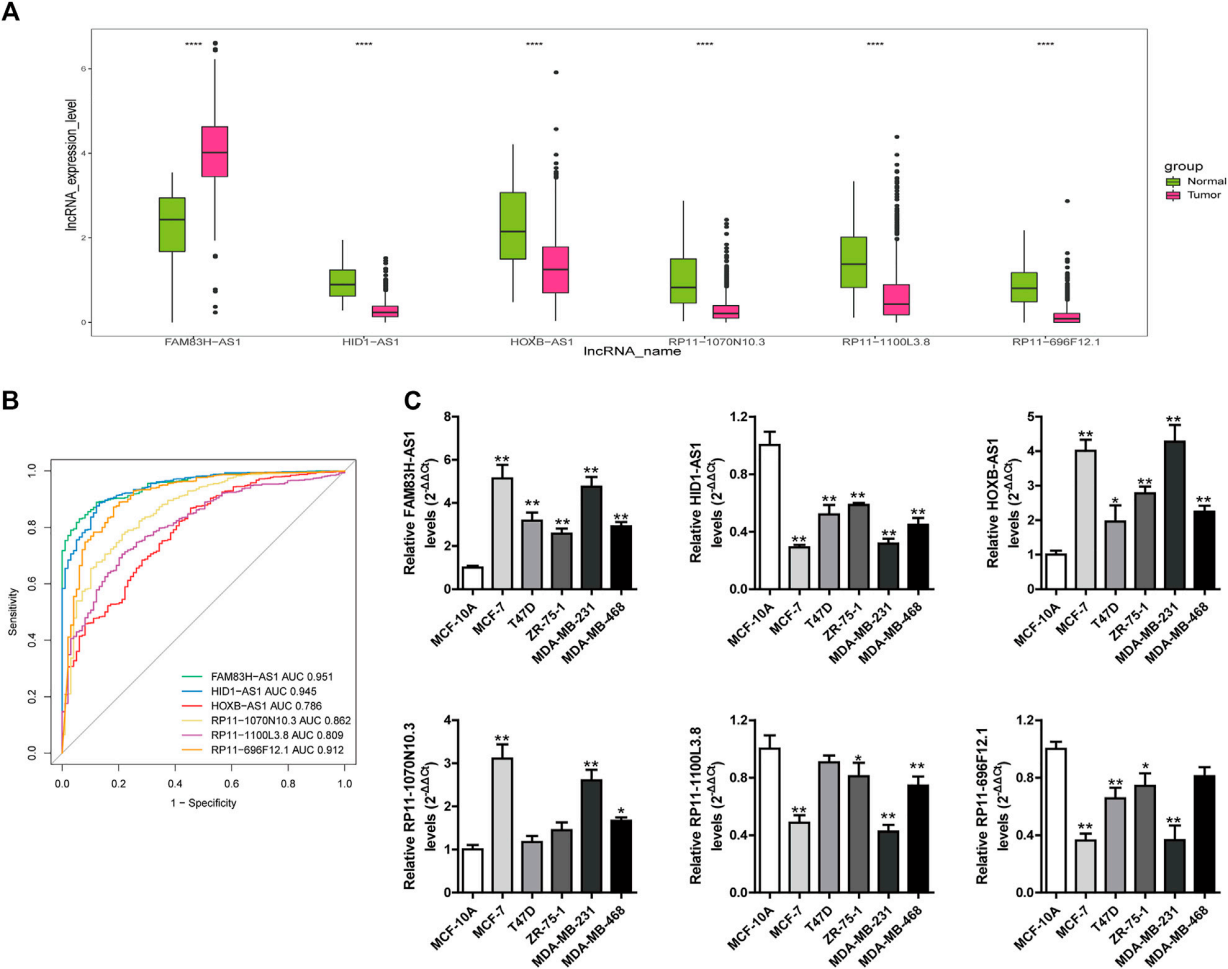
Investigation of diagnostic value of lncRNAs in stemness-index-related lncRNA signature. Expression levels of these lncRNAs in stemness-index-related lncRNA signature in TCGA database **(A)**, and ROC curves to evaluate their capability in distinguishing BC and normal samples in TCGA database **(B)**. Validation of expression of lncRNAs in stemness-index-related lncRNA signature by quantitative real-time polymerase chain reaction **(C).** Results were shown as mean ± SD. *p < .05 **p < .01 *vs*. MCF-10A.

To further explore whether these six lncRNAs in the stemness-index-related lncRNA signature can distinguish BC samples and normal samples, we firstly investigated the expression levels of these lncRNAs in the stemness-index-related lncRNA signature in TCGA database. As shown in Figure 7A, FAM83H-AS1 was upregulated in BC samples compared with normal samples, but HID1-AS1, HOXB-AS1, RP11-1070N10.3, RP11-1100L3.8, and RP11-696F12.1 were downregulated in BC samples compared with normal samples. Furthermore, ROC curves suggested that all of FAM83H-AS1, HID1-AS1, HOXB-AS1, RP11-1070N10.3, RP11-1100L3.8, and RP11-696F12.1 could distinguish recurrent BC and normal samples in TCGA database (Figure 7B). Thus, FAM83H-AS1, HID1-AS1, HOXB-AS1, RP11-1070N10.3, RP11-1100L3.8, and RP11-696F12.1 might be used as the diagnostic markers of BC.

### Cell Culture, RNA Isolation and Quantitative Real-Time Polymerase Chain Reaction

To further validate the expression level of FAM83H-AS1, HID1-AS1, HOXB-AS1, RP11-1070N10.3, RP11-1100L3.8, and RP11-696F12.1, we performed quantitative real-time PCR to detect their expression levels of them. Consistent with TCGA results, we found that FAM83H-AS1 were significantly upregulated in BC cells compared with normal cells, and HID1-AS1, RP11-1100L3.8, and RP11-696F12.1 were significantly downregulated in BC cells compared with normal cells (Figure 7C). However, the expression of HOXB-AS1, RP11-1070N10.3 were upregulated in BC cells compared with normal cells, which were in contrast to TCGA results. Therefore, FAM83H-AS1, HID1-AS1, RP11-1100L3.8, and RP11-696F12.1 could be used as the diagnostic biomarkers of BC.

## Discussion

BC is characterized as a highly heterogeneous disease, and it can be manifested by their classification into a number of distinct subtypes, each with a characteristic transcriptome and molecular expression signature (Visvader, 2009). Multiple evidence suggested that BC is organized and driven by a small number of tumor cells that display the characteristics of stem cells (Wicha et al., 2006). Once these cells are stimulated in some cases, they will get the ability to switch between a quiescent state and a proliferative state (Wicha et al., 2006; Charafe-Jauffret et al., 2008; Liu and Wicha, 2010). The presence of these stem cells is also associated with tumor survival, metastasis, and treatment resistance (Nassar and Blanpain, 2016). Although the studies on BC stem cells have been deepened worldwide, the role of stemness-index-related lncRNAs in the pathogenesis and progression of BRCA is unclear.

Our research aims to identify lncRNAs related to BC stemness index (mRNAsi and EREG-mRNAsi) by performing WGCNA. Through univariate and LASSO Cox regression analysis, we obtained six prognosis lncRNAs (FAM83H-AS1, HID1-AS1, HOXB-AS1, RP11-1070N10.3, RP11-1100L3.8, and RP11-696F12.1). Subsequently, we constructed a stemness-index-related lncRNA signature to predict the OS of BC patients based on the expression levels and corresponding coefficients derived from the LASSO Cox regression analysis. Moreover, we found that the stemness-index-related lncRNA signature could effectively predict the prognosis of BC patients and can be used as an independent prognostic factor in BC. Furthermore, we further explored the correlation between FAM83H-AS1, HID1-AS1, HOXB-AS1, RP11-1070N10.3, RP11-1100L3.8, RP11-696F12.1, and protein-coding genes in BC, separately. Interestingly, we found that FAM83H-AS1, HID1-AS1, HOXB-AS1, RP11-1070N10.3, RP11-1100L3.8, and RP11-696F12.1 may be involved in neuroactive ligand–receptor interaction, AMPK signaling pathway, PPAR signaling pathway, and cGMP-PKG signaling pathway. Finally, quantitative real-time PCR revealed that FAM83H-AS1, HID1-AS1, RP11-1100L3.8, and RP11-696F12.1 might be used as the potential diagnostic biomarkers of BC.

FAM83H-AS1, also known as onco-lncRNA-3, is located on chromosome 8 (8q24.3) and consists of 2,743 base pairs (Table 1). FAM83H-AS1 has been reported to act as an oncogene in several kinds of human cancers, such as cervical cancer (Barr et al., 2019), ovarian cancer (Dou et al., 2019), bladder cancer (Shan et al., 2019), glioma (Bi et al., 2018), rectal cancer (Lu et al., 2018), and lung cancer (Zhang et al., 2017). Consistent with our results, another research revealed that the expression of FAM83H-AS1 is increased and correlates with poor OS in patients with early-stage BC (Deva Magendhra Rao et al., 2019). Moreover, Han et al. (2020) also found that FAM83H-AS1 is associated with triple-negative BC progression by regulating miR-136-5p and MTDH expression. LncRNA HOXB-AS1 can promote the proliferation, migration, and invasion of glioblastoma cells (Bi et al., 2021), multiple myeloma (Chen R. et al., 2020), and endometrial carcinoma (Liu et al., 2020). However, it is less studied in BC. The only research is that lncRNA HOXB-AS1 may be related to N6-methyladenosine-(m6A)-mediated regulation in BC (Wu et al., 2021). As for other stemness-index-related lncRNAs, there are few studies related to tumorigenesis and progression, and more studies are needed.

**TABLE 1:** Primers used in quantitative polymerase chain reaction.

Primers	Sequence (5'→3′)	
FAM83H-AS1	Forward	5′-TAG​GAA​ACG​AGC​GAG​CCC-3′
	Reverse	5′-GCT​TTG​GGT​CTC​CCC​TTC​TT-3′
HID1-AS1	Forward	5′-GAG​CCA​TTT​CTG​TGG​CTT​GC-3′
	Reverse	5′-TGA​GTG​GTA​GAA​GAG​CCC​CT-3′
HOXB-AS1	Forward	5′-GGG​GAC​TCC​AGC​GAA​AT-3′
	Reverse	5′-ACC​CGA​AGC​CCA​ACC​AC-3′
RP11-1070N10.3	Forward	5′-ATG​AGC​GCT​ACT​AAT​GAA​GG-3′
	Reverse	5′-TAA​CCC​CGC​ATC​TGT​AAA​AT-3′
RP11-1100L3.8	Forward	5′-CTC​TGC​TGG​CAC​TTC​ACA​AA-3′
	Reverse	5′-CTC​GGG​TTC​TCA​CTT​GGA​GT-3′
RP11-696F12.1	Forward	5′-CTG​TTA​CCA​ACG​TCC​TAG​AG-3′
	Reverse	5′-TGA​CAA​TCA​CAC​ACT​TGG​AA-3′
GAPDH	Forward	5′-GGT​CTC​CTC​TGA​CTT​CAA​CA-3′
	Reverse	5′-GTG​AGG​GTC​TCT​CTC​TTC​CT-3′

The TME plays an important role in maintaining tumor stemness (Ye et al., 2014; Plaks et al., 2015). Notably, we found that the DEGs between the high-risk and low-risk groups were mainly involved in the immune-related biological processes and signaling pathways (Figure 6). It has been demonstrated that the interaction between tumor stem cells and their niche is closely related to the characteristics of tumor stem cells. Through this interaction, tumor stem cells can maintain tumor heterogeneity, which is the basis of important malignant biological behaviors such as invasion, metastasis, and therapeutic resistance (Dean et al., 2005; Beck and Blanpain, 2013). The components of TME are complex, and there are various types of cells in its niche, including endothelial cells, immune cells, tumor-related fibroblasts, and so on. In addition, various growth factors and cytokines in TME and hypoxia and pH changes are also important characteristics (Hjelmeland et al., 2011; Balkwill et al., 2012; Campos-Sánchez and Cobaleda, 2015). Therefore, we speculated that the stemness-index-related lncRNA signature might affect the CSC by regulating the composition of immune cells in the TME of BC.

In conclusion, our research identified and developed a novel stemness-index-related lncRNA signature for predicting the prognosis of BC patients based on six stemness-index-related lncRNAs (FAM83H-AS1, HID1-AS1, HOXB-AS1, RP11-1070N10.3, RP11-1100L3.8, and RP11-696F12.1). Moreover, we also found that stemness-index-related lncRNA signature is an independent prognostic factor and is related to the immune response. Finally, we also confirmed that FAM83H-AS1, HID1-AS1, RP11-1100L3.8, and RP11-696F12.1 might be used as the potential diagnostic biomarkers of BC. Thus, our results might provide a theoretical basis and reference value for improving the prognosis and diagnosis of BC, which may contribute to the clinic treatment of BC.

## Data Availability

The datasets presented in this study can be found in online repositories. The names of the repository/repositories and accession number(s) can be found in the article/Supplementary Material.
