# Supplementary figures and images for: Development and Validation of a Novel Stemness-Index-Related Long Noncoding RNA Signature for Breast Cancer Based on Weighted Gene Co-Expression Network Analysis
Source: Front Genet. 2022 Feb 22;13:760514. doi: 10.3389/fgene.2022.760514 (PMC8902307; doi:10.3389/fgene.2022.760514)

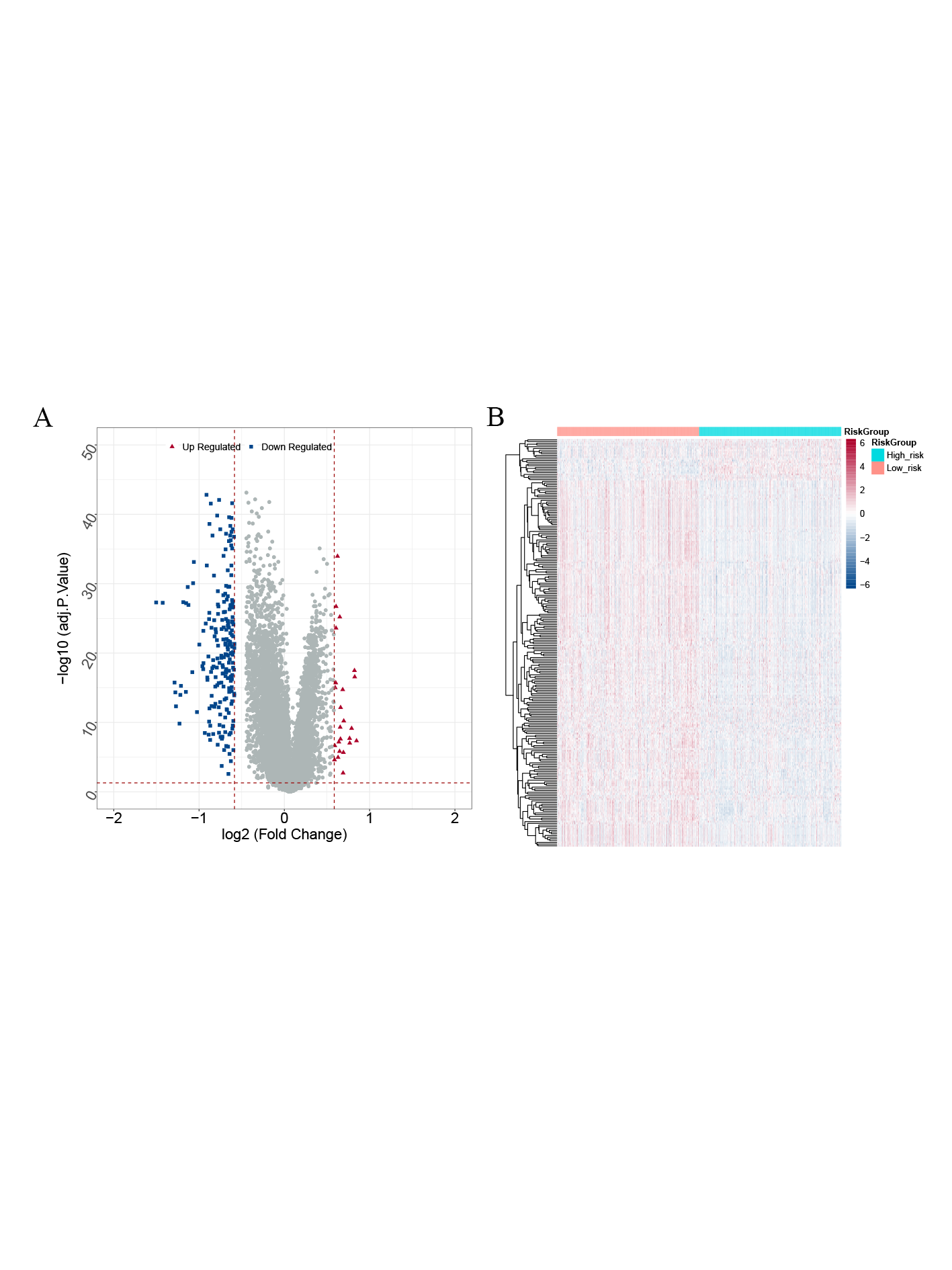

Supplement: Supplementary file 2 [file Image3.TIF]

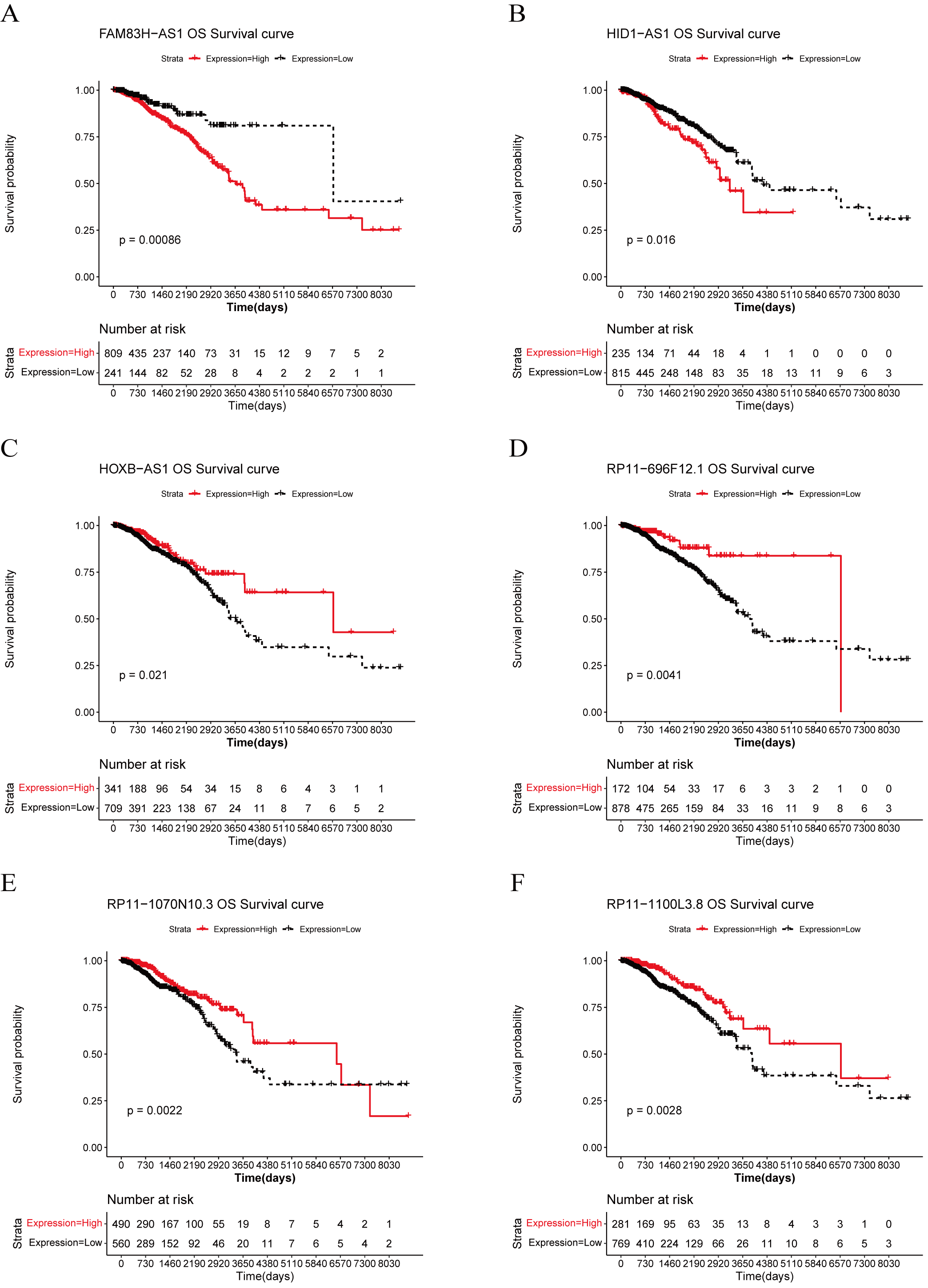

Supplement: Supplementary file 3 [file Image2.TIF]

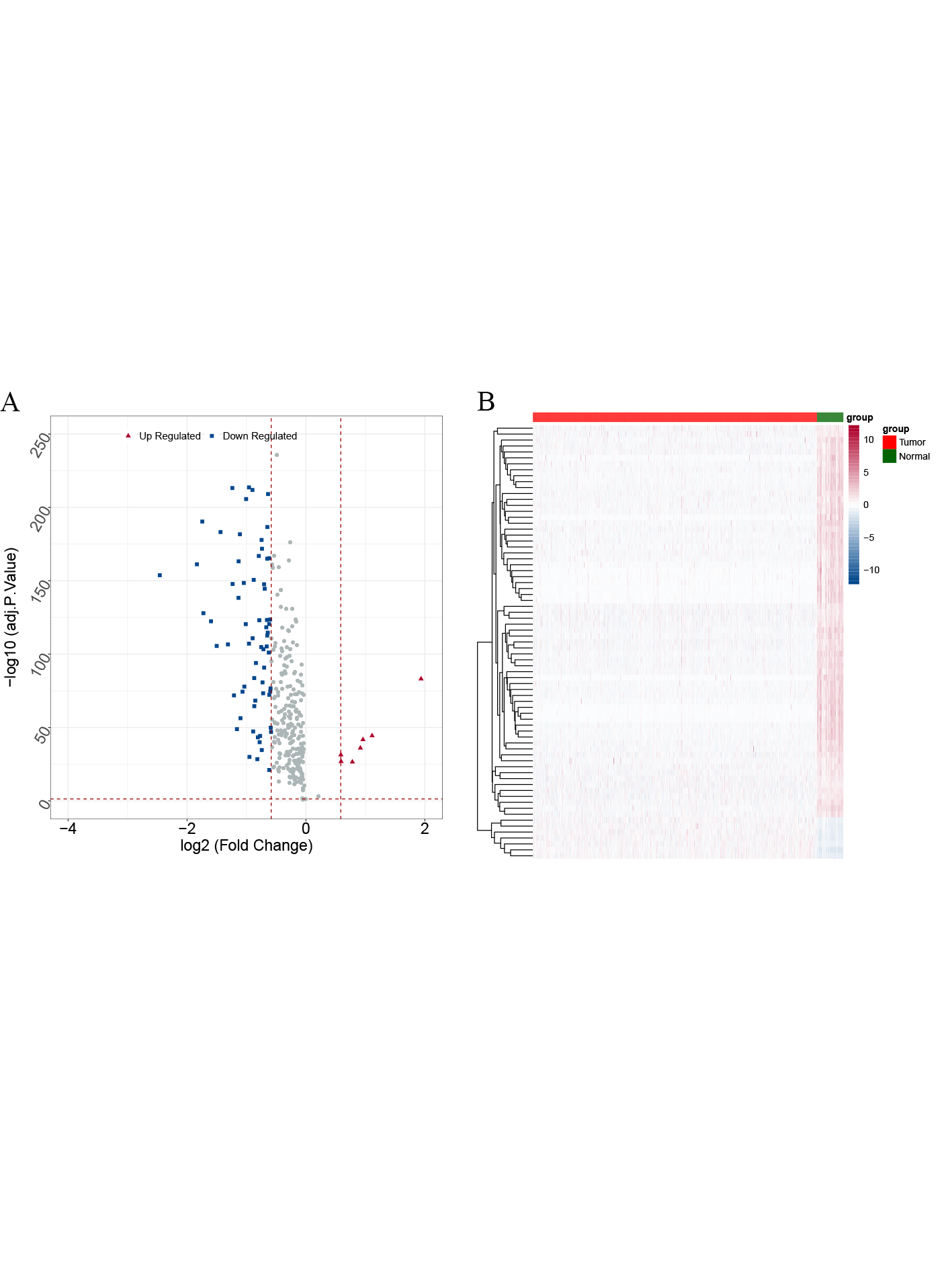

Supplement: Supplementary file 4 [file Image1.TIF]
